# The evolving landscape of gene editing therapies for human genetic diseases: a twenty-year bibliometric analysis

**DOI:** 10.3389/fmed.2026.1872028

**Published:** 2026-06-03

**Authors:** Xinhao Zhou, Tengfei Ma, Xu Ding, Zhenzhao Luo, Changqing Yin, Zhe Lu

**Affiliations:** 1 Department of Clinical Laboratory, Wuhan Fourth Hospital, Wuhan, Hubei, China; 2 Department of Medical Laboratory, The Central Hospital of Wuhan, Tongji Medical College, Huazhong University of Science and Technology, Wuhan, Hubei, China; 3 Department of Medical Laboratory, Jingmen Center Hospital / Affiliated Jingmen Center Hospital, Jingchu University of Technology, Jingmen, Hubei, China; 4 College of Life Sciences, Zhejiang University, Hangzhou, China; 5 Department of Transfusion, Wuhan Fourth Hospital, Wuhan, Hubei, China

**Keywords:** bibliometric analysis, CRISPR-Cas, gene editing, Gene Therapy, human genetic diseases

## Abstract

**Background/Objectives:**

Despite the transformative potential of gene editing technologies, a systematic mapping of their translational trajectory from bench to bedside remains scarce. This bibliometric analysis aims to chart the bench-to-bedside evolution, identify leading disease targets and therapeutic strategies, and uncover emerging clinical trends and challenges.

**Methods:**

We analyzed 1,571 peer-reviewed articles and reviews explicitly addressing gene editing therapies for human genetic diseases, published between 2005 and 2025 and retrieved from Web of Science Core Collection and Scopus. Science mapping was conducted using CiteSpace and VOSviewer to visualize collaboration networks, thematic clusters, and research fronts.

**Results:**

Publication output followed a three-phase exponential growth: engineered nucleases (2005–2012), CRISPR revolution (2013–2018), and precision translation (2019–2025). The United States and China dominated productivity; Harvard and the University of California were key institutional hubs. Hematologic disorders—particularly sickle cell disease and β-thalassemia—constituted the primary disease focus. Emerging frontiers include base and prime editing, epigenetic modulation, multiplex editing, and AI-assisted design. Collaboration networks remain predominantly national, with limited global integration.

**Conclusions:**

This study delineates the rapid evolution of gene editing therapies, highlighting robust clinical translation for hematological conditions while exposing critical gaps in non-hematopoietic tissues, delivery efficiency, long-term safety, and equitable access. These findings provide a strategic roadmap to broaden the therapeutic reach of gene editing across diverse disease domains.

## 1 Introduction

The ability to precisely modify genomic sequences stands as one of the most transformative advances in modern biology, yet a systematic understanding of its translational trajectory remains incomplete. Early genome manipulation relied on homologous recombination in embryonic stem cells—a process that was inefficient, time-consuming, and largely restricted to model organisms (1). The emergence of programmable nucleases marked the first generation of targeted genome editing, beginning with Zinc Finger Nucleases (ZFNs) in 1996 (2) and followed by Transcription Activator-Like Effector Nucleases (TALENs) in 2009 (3). Despite their precision, these protein-based systems required laborious engineering for each target site, limiting scalability and broad application.

A paradigm shift occurred with the discovery and adaptation of the CRISPR-Cas9 system. Originally identified as a bacterial adaptive immune mechanism by Mojica et al. in 2005 (4), CRISPR-Cas9 was repurposed for eukaryotic genome editing in 2012–2013 through landmark studies by Jinek et al. (5), Mali et al. (6), and Cong et al. (7). Its simplicity—relying on RNA-guided DNA targeting—democratized gene editing, catalyzing an explosion of research across functional genomics, disease modeling, and therapeutic development.

The programmable precision and ease of use of CRISPR-Cas9 have not only revolutionized basic research but also accelerated the translation of gene editing into clinical practice. This potential is particularly profound for monogenic disorders, where correcting the causative mutation offers a curative strategy. CRISPR-based therapies have rapidly progressed from concept to clinic, marked by pivotal milestones: the 2023 FDA and EMA approvals of exagamglogene autotemcel (Casgevy™) for sickle cell disease and β-thalassemia (8, 9); the development of next-generation tools such as base editing and prime editing, enabling precise single-nucleotide changes without inducing double-strand breaks (10, 11); and successful *in vivo* editing in conditions like transthyretin amyloidosis using lipid nanoparticle delivery (12). The transformative impact of this technology was recognized globally with the award of the 2020 Nobel Prize in Chemistry to Emmanuelle Charpentier and Jennifer A. Doudna (13). Together, these advances herald a new era in genetic medicine.

Despite rapid progress, critical knowledge gaps persist in understanding the translational landscape of gene editing for human genetic diseases. While narrative reviews are abundant (14, 15), there remains a lack of systematic, quantitative mapping of the field's two-decade evolution. Bibliometric analysis offers a powerful tool to uncover influential works, collaboration networks, and thematic trends (16, 17). Yet, existing bibliometric studies on CRISPR often focus on narrow timeframes or specific applications, failing to capture the full scope of the field's development and recent clinical advances.

To address these limitations, this study presents a comprehensive bibliometric analysis of gene editing therapy research from 2005 to 2025. We aim to: (i) quantify publication trends and growth dynamics; (ii) map global and institutional contributions through collaboration network analysis; (iii) identify key authors, journals, and foundational literature; and (iv) detect research hotspots and evolutionary trajectories using keyword co-occurrence and burst detection. By delineating developmental phases and forecasting emerging frontiers, this work provides evidence-based insights to guide researchers, funding agencies, and policymakers in shaping the future of genetic therapeutics. Notably, recent bibliometric analyses have focused on specific delivery systems (18, 19). Our study extends these by integrating 20-year trends, dual databases (WoSCC and Scopus), and a specific focus on therapeutic translation for human genetic diseases.

## 2 Materials and methods

### 2.1 Database selection

To construct a robust and representative dataset, we employed a dual-database strategy integrating the Web of Science Core Collection (WoSCC) and Scopus. This approach effectively mitigates the coverage limitations inherent to any single repository. WoSCC serves as the cornerstone of our analysis, prized for its stringent journal selection and precise citation tracking, which are indispensable for accurate bibliometric mapping (20, 21). To address potential gaps, Scopus was incorporated for its extensive coverage of life science disciplines and non-Western publications. By synthesizing the depth of WoSCC with the breadth of Scopus, we ensure a holistic view of the field, thereby reinforcing the reliability and generalizability of our conclusions.

### 2.2 Search strategy

**Figure 1:**
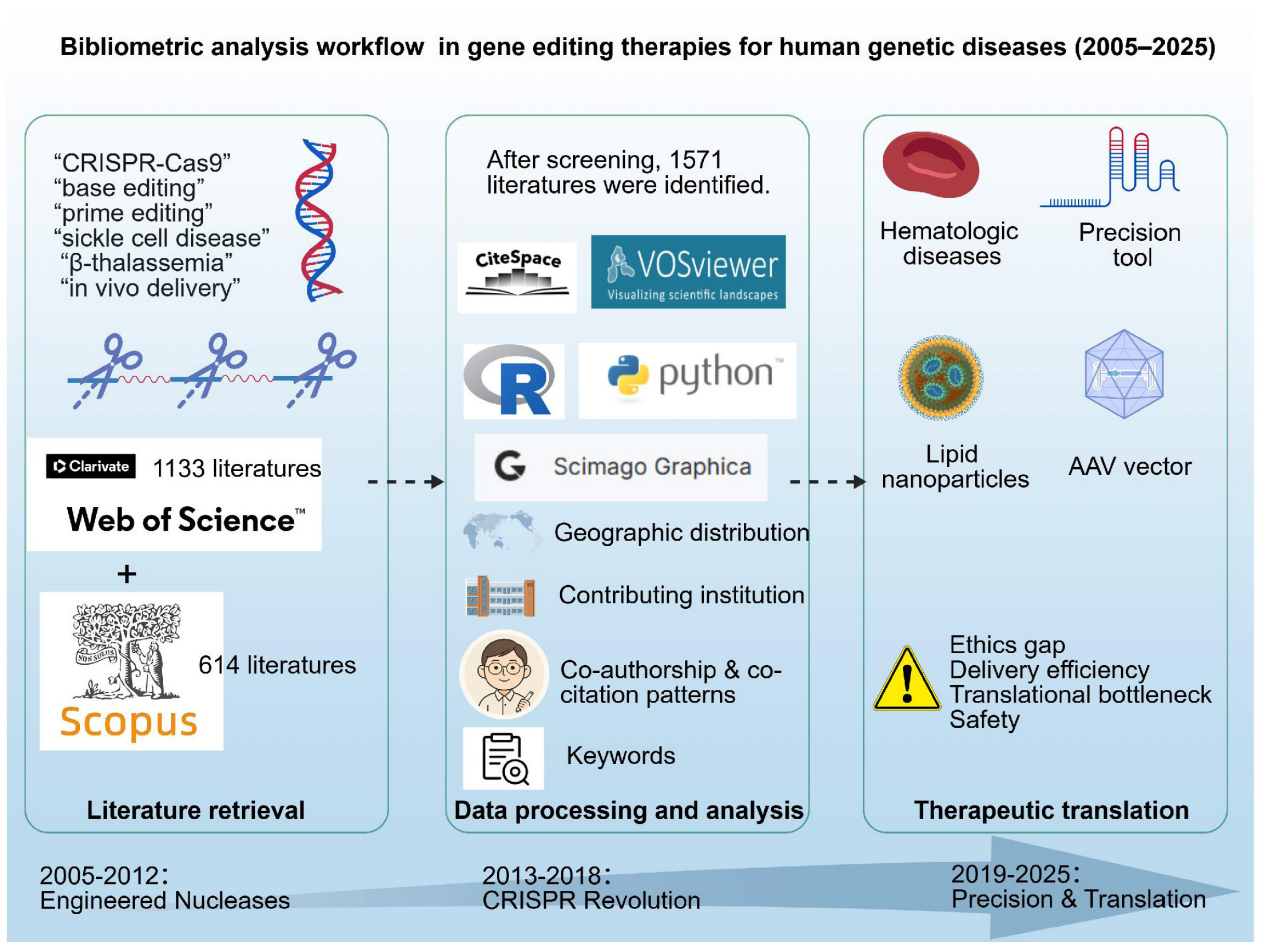
Flow diagram of study selection and data analysis strategies (Created with BioGDP.com) (71).

As depicted in Figure 1, a comprehensive literature search was performed using the Advanced Search modules of WoSCC and Scopus. The finalized search protocols, executed on December 31, 2025, are listed in Supplementary Tables 1, 2. Data extraction was confined to English-language publications classified as “articles” or “reviews” within the timeframe of January 1, 2005, to December 31, 2025. This search strategy, excluding computational studies (particularly AI-driven CRISPR design), has inadvertently narrowed the scope of our review.

### 2.3 Data selection and standardization

To ensure the relevance, quality, and suitability of the dataset for bibliometric analysis, literature was rigorously screened using predetermined inclusion and exclusion criteria. Inclusion criteria encompassed peer-reviewed articles and reviews published in English between January 1, 2005, and December 31, 2025, with a primary focus on gene/genome editing technologies, their underlying mechanisms, or specific applications. Conversely, to maintain analytical precision, the following categories were excluded: (1) studies discussing CRISPR systems solely in the context of native bacterial immunity without reference to engineered editing applications; (2) purely computational or bioinformatics studies focused on CRISPR array prediction lacking experimental validation; (3) non-primary document types (e.g., editorials, letters, meeting abstracts, and corrections); (4) retracted publications to uphold data integrity; and (5) records with incomplete critical metadata (e.g., missing author or institutional affiliations) that would preclude network and institutional analyses. Two independent reviewers (X.Z. and T.M.) screened titles and abstracts against the predefined criteria, with discrepancies resolved through consultation with a third reviewer (X.D.). Following the merging of initial records, removal of duplicates, and application of exclusion criteria, complete bibliographic information was exported in plain text format. This dataset includes authors, titles, abstracts, keywords, journal details, institutional affiliations, countries, cited references, and citation counts (as of December 31, 2025).

### 2.4 Bibliometric analysis and quality assurance

The analysis was conducted using several complementary bibliometric software packages to leverage their respective strengths. Python was used for data preprocessing. VOSviewer (version 1.6.20) (22) was primarily employed for constructing and visualizing collaborative networks (co-authorship analysis at author, institutional, and country levels), co-citation networks and keyword co-occurrence. Key network metrics, including total link strength and cluster density, were calculated within this environment. For visualization, minimum document thresholds were set (5 for authors, 20 for institutions, 50 for countries). To capture the dynamic and structural intricacies of the field, CiteSpace (version 6.4.R1) (23) was employed with the following configuration: time slicing in 1-year intervals across 2005–2025, node types (e.g.keywords) selected according to each specific analysis. Pathfinder network scaling and Pruning Sliced Networks were applied to trim redundant links and bring the most salient connections to the fore. Within this framework, dual-map overlays were generated to trace interdisciplinary citation flows between citing and cited journal clusters, and Kleinberg's burst detection algorithm (24) was used to identify keywords that exhibited a sudden surge in frequency over a defined period, flagging emergent research fronts. Timeline views were constructed to follow the evolution of co-citation clusters, and betweenness centrality was computed to pinpoint pivotal nodes that structurally bridge different areas of the literature. R and Scimago Graphica Beta 1.0.51 were utilized for visualizing the analytical findings. Detailed methodological parameters, including deduplication logic and software justification, are provided in Supplementary Appendix A.

## 3 Results

### 3.1 . Publication trends and growth dynamics

**Figure 2:**
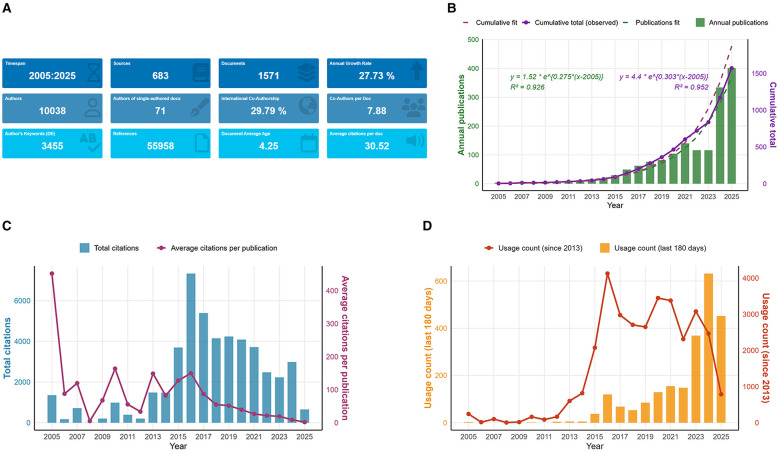
The annual and cumulative number of publications from 2005 to 2025. **(A)** The overview of included literature. **(B)** Number of publications per year and growth trend of publications. **(C)** Total citations of publications per year and average citations per publication per year. **(D)** Number of publications usage count in the last 180 days per year and number of publications usage count since 2013 per year.

Figure 2A provides a general overview of the bibliometric analysis conducted in this study. As illustrated in Figure 2B, the temporal evolution of publications on gene editing therapies for human genetic diseases can be descriptively categorized into three *post-hoc* phases.. The field experienced a nascent phase (2005–2012), followed by a rapid acceleration phase (2013–2019) triggered by the CRISPR-Cas9 breakthrough, and has now entered a period of sustained expansion (2020–2025). Notably, the last 6 years account for 77.3% of all recorded publications (*n* = 1,214), quantitatively marking this as the field's most prolific era. The exponential model demonstrates an excellent fit for both annual (R^2^ = 0.926) and cumulative (R^2^ = 0.952) data. This growth corresponds to an annual growth rate (AGR) of 27.73%, with a publication doubling time of just 2.5 years. Figure 2C illustrates the temporal distribution of citation impact. Total citations per year exhibited a substantial increase post-2015, rising from 3,696 in 2015 to a peak of 7,337 in 2016, following relatively high early counts (e.g., 1,355 in 2005 and 981 in 2010). In contrast, the average citations per publication displayed a declining trend over time, with early publications achieving notably high averages (e.g., 451.7 in 2005), while recent articles, such as those from 2025, averaged only 1.6 citations due to limited citation windows. The high citation counts for early publications (e.g., 2005) are directly linked to landmark papers that established the foundational repair mechanisms, such as the work by Capecchi (1). The subsequent spike in total citations around 2016 corresponds to the highly influential methodology papers on CRISPR specificity [e.g., (33, 34)] which paved the way for therapeutic applications. Thus, the Figure not only reports data but critically interprets these trends in the context of specific milestone publications. Figure 2D reveals a consistent upward trajectory in both recent (last 180 days) and cumulative usage counts since 2013, indicating a marked increase in scholarly engagement and practical application. Collectively, these metrics suggest that the field entered a phase of accelerated development around 2013, characterized by rapid growth in publication volume, citation frequency, and research utilization.

### 3.2 National contributions

**Figure 3:**
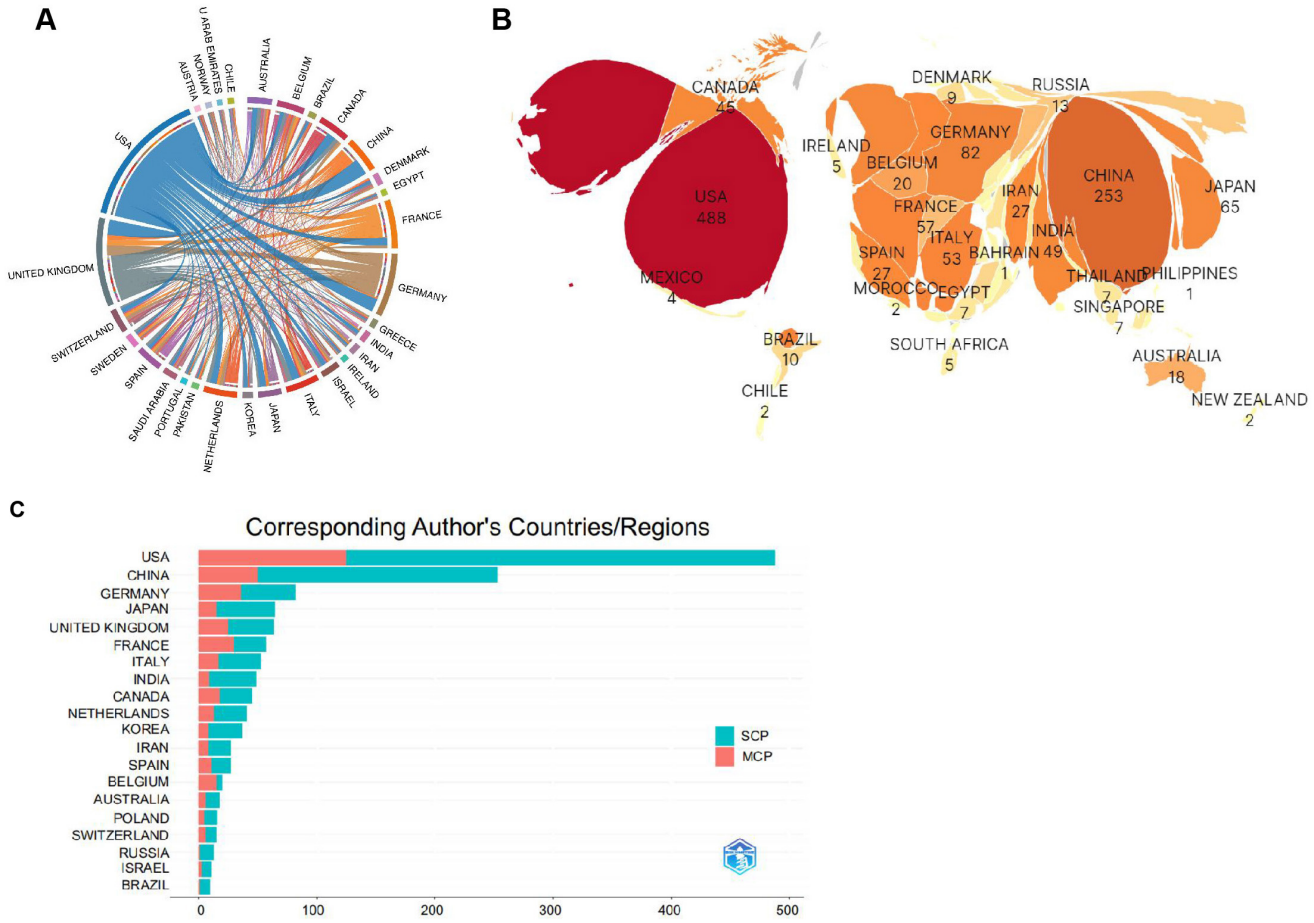
Geographic distribution of publications in the field of gene editing within human genetic diseases, based on bibliometric data. **(A)** Chord diagram showing national/regional collaboration networks. **(B)** Cartogram displaying publication volume and collaboration patterns. **(C)** The top 20 countries/regions of corresponding authors ranked by number of publications. SCP, single country publications; MCP, multiple country publications.

A total of 85 countries/regions contributed to publications in the field of gene editing within human genetic diseases research, as measured by corresponding author appearances per country. Analysis of global contributions reveals a concentrated yet stratified research landscape, wherein the top 15 countries accounted for 84.4% of total output, indicating a pronounced concentration of research productivity in high-income nations (Table 1). The United States maintains definitive leadership, evidenced by its foremost position in publication volume (488 articles), total citations (27,508), and total link strength (482), solidifying its status as the central hub for innovation in this domain (25). While China ranks second in output (253 publications), its notably lower average citation rate compared to the U.S. suggests an area for maturation in research impact; however, its exceptional annual publication growth rate (31.95% from 2015–2020), the highest among all contributing nations, aligns with its documented surge in R&D investment (26). In contrast, key European nations—Germany, the United Kingdom, France, and the Netherlands—collectively contributed 15.5% of publications and are distinguished by their high average citations (ranging from 22.85 to 51.62), reflecting a strong, quality-oriented research culture often supported by coordinated funding mechanisms such as the European Research Council (27). Figures 3A, 3B shows the geographic distribution and collaboration network of publications in the field of gene editing within human genetic diseases, Figure 3C displays the top 20 countries/regions ranked by the number of publications with corresponding authors. The US, China, Germany, Japan, and the UK lead this list. With 363 single country publications (SCP) and 125 multiple country publications (MCP), the US holds the highest counts in both categories. These figures underscore its dominant role in this research domain.

**Table 1:** Top 15 countries/regions ranked by publication output.

Rank	Country	Docs	% Total	Citations	Total link strength
1	USA	488	31.06%	27508	482
2	China	253	16.10%	4306	114
3	Germany	82	5.22%	1828	220
4	Japan	65	4.14%	1244	80
5	UK	64	4.07%	1583	296
6	France	57	3.63%	1032	171
7	Italy	53	3.37%	710	113
8	India	49	3.12%	607	41
9	Canada	45	2.86%	508	106
10	Netherlands	41	2.61%	2065	115
11	South Korea	37	2.36%	855	31
12	Spain	27	1.72%	590	91
13	Iran	27	1.72%	385	38
14	Belgium	20	1.27%	636	78
15	Australia	18	1.15%	308	88

### 3.3 Institutional contributions

**Figure 4:**
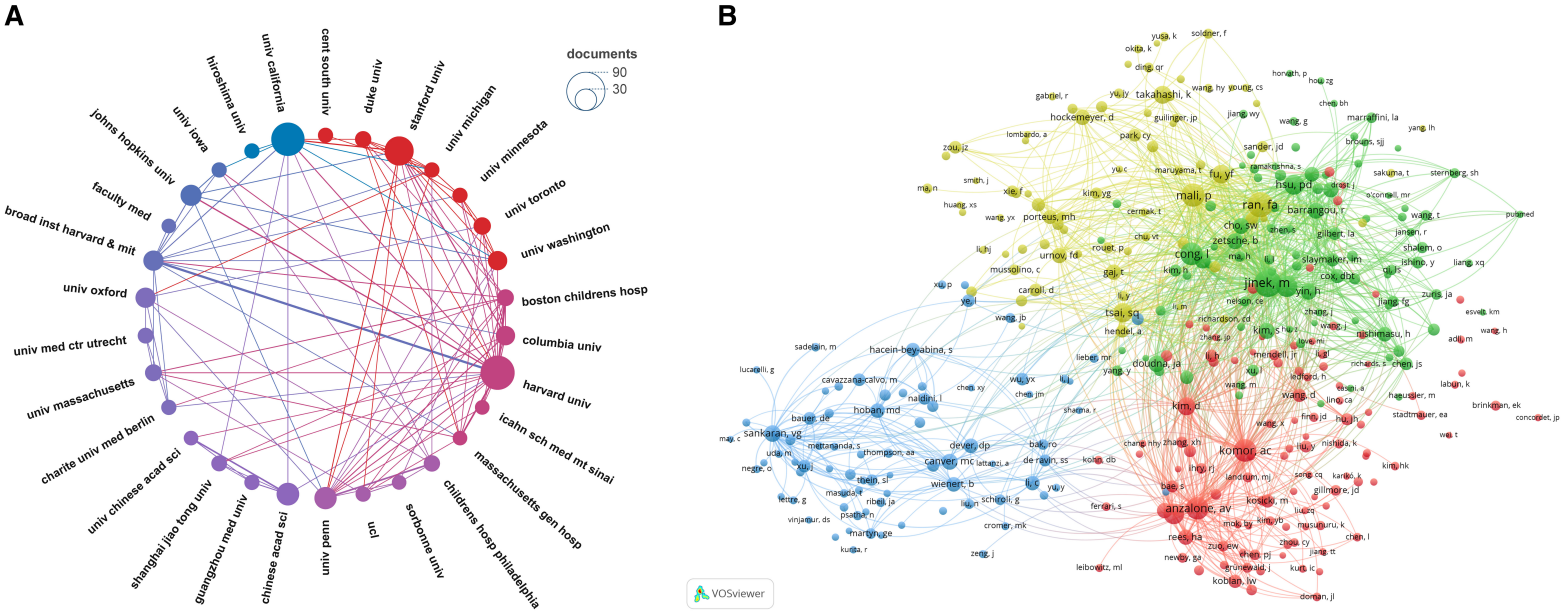
Networks of collaboration on gene editing within human genetic diseases. **(A)** Institutional collaboration. **(B)** Author collaboration.

Among 843 institutions globally,6 published ≥20 papers. The top 20 institutions produced 323 papers (20.56% of total), demonstrating significant concentration consistent with the Matthew effect in scientific research (28) (Table 2). Harvard University emerges as the leading hub of innovation, leading with 66 publications and amassing 10,952 citations, which underscores its seminal influence in the field. This leadership anchors a broader pattern of U.S. institutional dominance, as 12 of the top 15 most prolific organizations are American, collectively contributing 357 publications or 22.72% of the global output—a direct reflection of concentrated research capital from sources such as the National Institutes of Health and substantial venture funding (29). Concurrently, the Chinese academic system demonstrates formidable capacity through a centralized, state-coordinated model; the Chinese Academy of Sciences ranks fourth with 29 publications, and when combined with other leading institutions such as Peking University, Tsinghua University, and the Shanghai Institutes, the collective output reaches 85 papers, representing 5.4% of the total and highlighting the strategic impact of national initiatives like the National Key R&D Program. Additionally, the top 30 institutions by publication count are presented in Figure 4A.

**Table 2:** Top 15 institutions by publication output.

Rank	Institution	Country	Docs	Citations	Total link strength
1	Harvard University	USA	66	10952	137
2	University of California	USA	63	7893	51
3	Stanford University	USA	48	3969	67
4	Chinese Academy of Sciences	China	29	791	46
5	University of Pennsylvania	USA	28	1089	53
6	Johns Hopkins University	USA	25	678	28
7	Broad Institute of MIT and Harvard	USA	23	4021	61
8	University of Oxford	UK	23	1159	28
9	Columbia University	USA	21	949	57
10	University of Washington	USA	21	305	18
11	University of Toronto	Canada	17	280	29
12	Boston Children's Hospital	USA	16	1099	58
13	University of Massachusetts	USA	16	894	23
14	Children's Hospital of Philadelphia	USA	15	804	27
15	Duke university	USA	15	1157	15

### 3.4 Prolific authors

Analysis of author contributions reveals distinct productivity and impact profiles among leading researchers. A total of 10,168 authors have participated in publishing research on gene editing in human genetic diseases. As shown in Table 3, leading the field by volume is Nakamura, Yukio, with 14 publications, 679 citations, and a total link strength of 17. Authors with identical publication counts, such as Kurita, Ryo (11 documents, 657 citations) and Bauer, Daniel E. (11 documents, 1484 citations), exhibit markedly divergent citation impacts, highlighting the variable influence of individual contributions. This pattern is further underscored by Liu, David R., whose 11 publications have accrued an exceptional 7,433 citations, despite a relatively modest total link strength of 5, indicating that a high-impact publication can disproportionately drive scholarly recognition. Among authors ranked fifth to tenth (with 7–10 publications each), Hu, Zhiqing and Zhou, Miaojin demonstrate the strongest collaborative networks (total link strength of 18 each), yet their citation counts (113 each) remain comparatively low. Collectively, these metrics illustrate the complex and non-linear relationship between publication productivity, citation-based impact, and network centrality within this research domain (Figure 4B). This decoupling suggests that productivity alone is an insufficient predictor of an author's scholarly influence or collaborative reach.

**Table 3:** Top 10 authors by publication count.

Rank	Author	Docs	Citations	Total link strength
1	Nakamura, Yukio.	14	679	17
2	Kurita, Ryo.	11	657	17
3	Bauer, Daniel E.	11	1484	9
4	Liu, David R.	11	7433	5
5	Porteus, Matthew H.	10	2440	10
6	Miccio, Annarita	9	468	11
7	Yamamoto, Takashi	8	712	10
8	Hu, Zhiqing	7	113	18
9	Zhou, Miaojin	7	113	18
10	Bao, Gang	7	958	8

### 3.5 Journal distribution and impact

**Figure 5:**
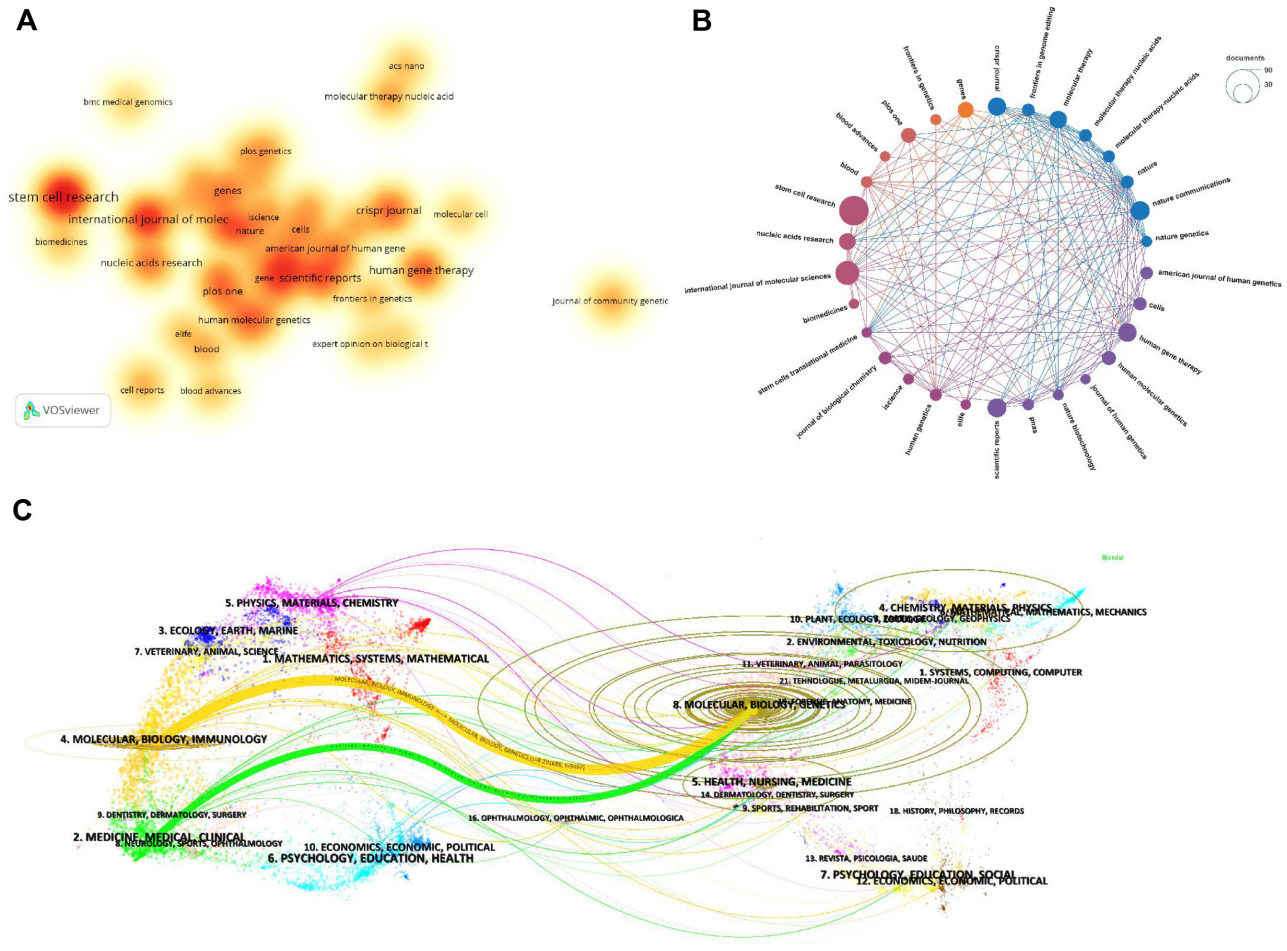
Analysis of journals related to gene editing within human genetic diseases. **(A)** Map of journals with ≥ 5 publications. **(B)** Citation map of the 30 core journals identified by Bradford's law of scattering; **(C)** Dual-map overlay analysis of journals.

Gene editing therapy research for human genetic disorders was published across 684 journals. The top 15 journals accounted for 22.8% (358 papers) of total output (Table 4). The analysis of journal influence reveals a clear decoupling between productivity and prestige. Journals characterized by high output volume, exemplified by Stem Cell Research, often occupy a peripheral position in the knowledge network. This is manifested in their lower JCR quartile ranking and, more critically, in their minimal Total Link Strength within the co-citation network, indicating limited integration into the field's core discursive flow. In stark contrast, elite multidisciplinary journals such as Nature and Nature Communications exert a disproportionately high influence. Despite lower publication counts, their articles achieve exceptional citation rates and dominate the co-citation network through high Total Link Strength, solidifying their role as primary knowledge hubs. Furthermore, the research output is concentrated in high-quality venues. Among the leading journals, the majority (9 out of 15) are ranked in JCR Q1, including specialized high-impact titles like Molecular Therapy and Nucleic Acids Research. Notably, a subset of these Q1 journals—including International Journal of Molecular Sciences, Nature Communications, Human Gene Therapy, and Molecular Therapy—exhibits strong mutual connectivity (Total Link Strength). This pattern suggests they constitute a core, interactive knowledge cluster that channels and structures the principal scholarly discourse, effectively setting the agenda for the field's development. Figure 5A depicts the 59 journals that contributed five or more publications. In the visualization, font size corresponds to publication output—journals with higher counts are represented by larger names—while chromatic intensity reflects increasing publication volume through darker red shading. Applying Bradford's law of scattering, a total of 30 core journals were identified in the field of gene editing therapies for human genetic diseases (Figure 5B).

**Table 4:** Top 15 journals by publication count.

Rank	Journal	Papers	IF (2024)	JCR	Citations	Total link strength
1	Stem Cell Research	66	0.7	Q4	135	1
2	International Journal of Molecular sciences	43	4.9	Q1	959	64
3	Nature Communications	27	15.7	Q1	1107	68
4	Scientific Reports	27	3.9	Q1	353	17
5	Human Gene Therapy	26	4.0	Q1	494	68
6	Crispr Journal	25	4.0	Q1	152	31
7	Molecular Therapy	23	12.0	Q1	1006	84
8	Nucleic Acids Research	21	13.1	Q1	489	36
9	Genes	19	2.8	Q2	232	24
10	PLOS ONE	17	2.6	Q2	160	16
11	Human Molecular Genetics	15	3.2	Q2	546	13
12	Cells	13	5.2	Q2	178	28
13	Nature	12	48.5	Q1	9853	233
14	Frontiers in Genome Editing	12	4.4	Q1	158	60
15	Journal of Biological Chemestry	12	3.9	Q2	486	22

CiteSpace was employed to generate a dual-map overlay of journals, visualizing citation relationships between citing (left) and cited (right) journal clusters. As illustrated in Figure 5C, two predominant citation trajectories were identified—one yellow and one green path. Notably, both trajectories converge on journals within the Molecular/Biology/Genetics cluster. This pattern indicates that research published in journals categorized under Molecular/Biology/Immunology, Medicine/Medical/Clinical, and Neurology/Sports/Ophthalmology frequently cites foundational literature from Molecular/Biology/Genetics journals.

### 3.6 Most influential papers

**Figure 6:**
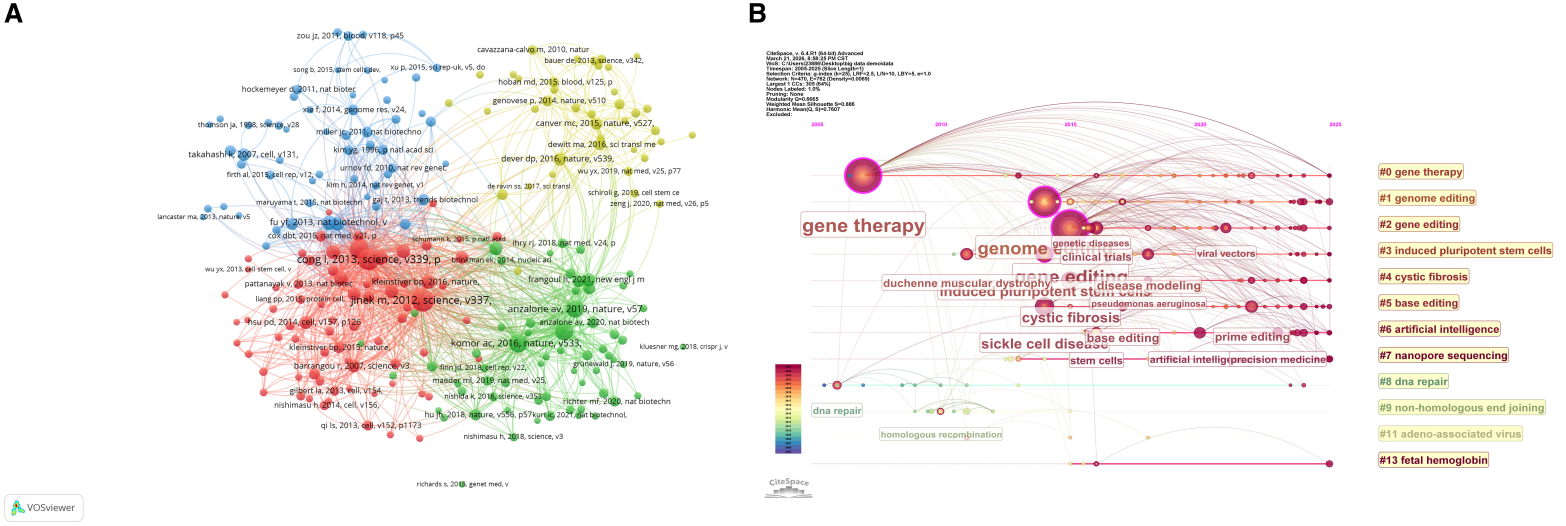
Co-citation analysis of influential publications. **(A)** Network visualization of co-cited references. **(B)** Timeline visualization of co-citation clusters.

Co-citation analysis revealed the ten most influential publications that have fundamentally defined the intellectual foundation of the field (Table 5). These seminal works collectively delineate three pivotal advancements: the elucidation of the core CRISPR-Cas9 mechanism (5–7, 30, 31), which established the paradigm of RNA-guided DNA targeting (5–7, 30, 31); the subsequent development of precision editing tools—including base editors (10, 32) and prime editors (11)—that enabled targeted nucleotide conversions without requiring double-strand breaks (DSBs) (10, 11, 32) and critical innovations aimed at enhancing specificity (33, 34), which significantly mitigated off-target effects and paved the way for therapeutic applications (33, 34). The network analysis revealed a distinct modular structure comprising four major thematic clusters, each color-coded in Figure 6A. The red cluster encompasses the foundational literature on the CRISPR/Cas mechanism, while the green cluster is focused on precision genome editing tools. Complementary to these, the blue cluster centers on enhancing nuclease specificity, and the yellow cluster aggregates research applying these technologies to human genetic diseases. The temporal evolution of these research fronts, illustrated in the timeline view (Figure 6B), demonstrates a clear knowledge progression: clusters related to core mechanisms (Clusters #1, #2) peaked in activity between 2013 and 2015, followed by the emergence and growth of precision editing themes (Clusters #5, #7) from 2016 onward. Notably, clusters associated with clinical translation and delivery strategies (Cluster #11, #13) have become the dominant research frontier in the most recent period (2021–2025 within the analyzed dataset).

**Table 5:** Top 10 most co-cited references.

Rank	First Author	Year	Title	Journal	Co-citations
1	Jinek M	2012	A programmable dual-RNA-guided DNA endonuclease in adaptive bacterial immunity	Science	214
2	Cong L	2013	Multiplex genome engineering using CRISPR/Cas systems	Science	195
3	Mali P	2013	RNA-guided human genome engineering via Cas9	Science	151
4	Komor AC	2016	Programmable editing of a target base in genomic DNA without double-stranded DNA cleavage	Nature	148
5	Gaudelli NM	2017	Programmable base editing of A•T to G•C in genomic DNA without DNA cleavage	Nature	134
6	Anzalone AV	2019	Search-and-replace genome editing without double-strand breaks or donor DNA	Nature	126
7	Ran FA	2013	Genome engineering using CRISPR-Cas9 system	Nature Protocols	98
8	Fu YF	2013	High-frequency off-target mutagenesis induced by CRISPR-Cas nucleases in human cells	Nature Biotechnology	85
9	Kleinstiver BP	2016	High-fidelity CRISPR-Cas9 nucleases with no detectable genome-wide off-target effects	Nature	82
10	HSU PD	2013	DNA targeting specificity of RNA-guided Cas9 nucleases	Nature Biotechnology	82

### 3.7 Keyword analysis and research hotspots

**Figure 7:**
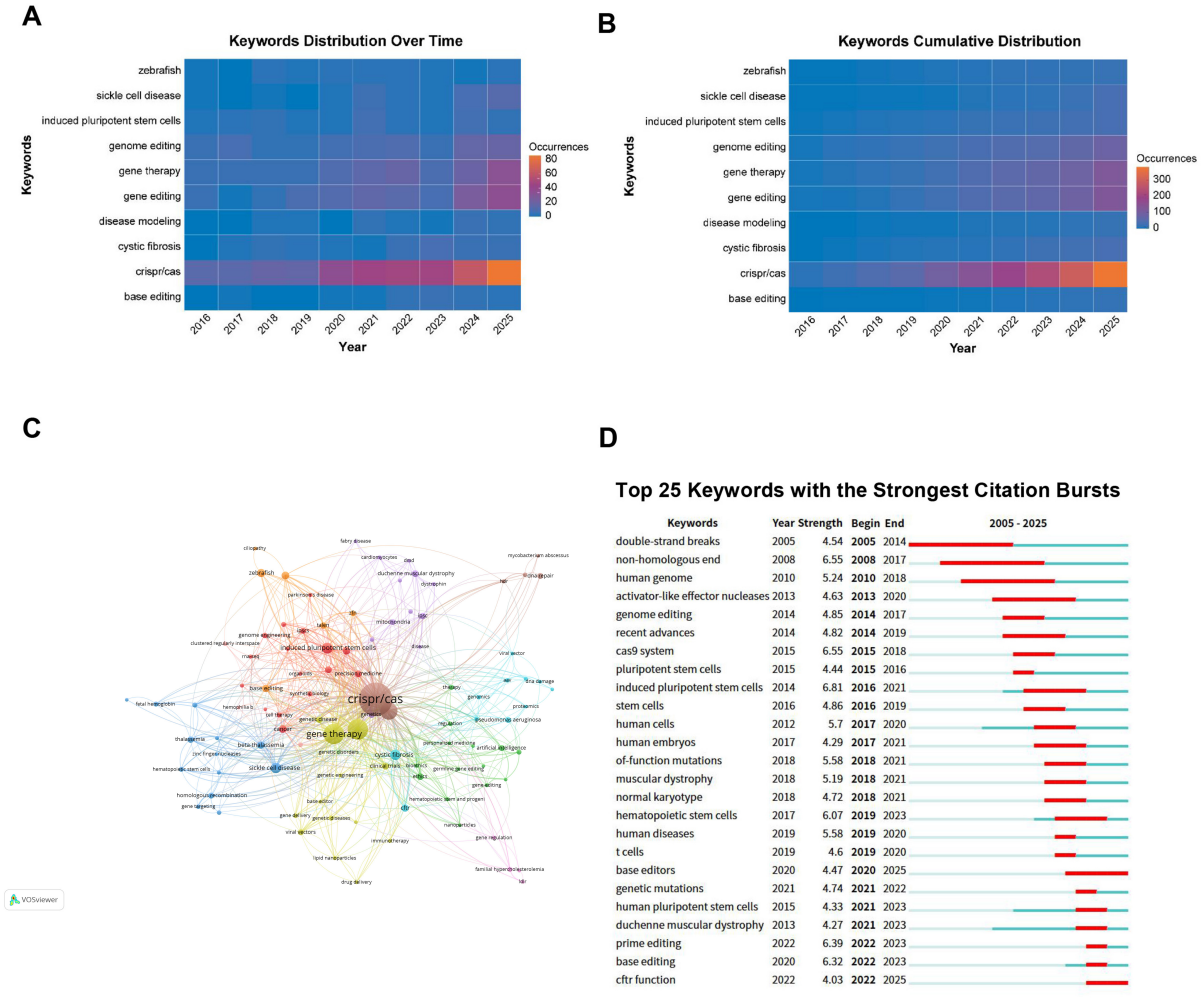
Analysis of keywords on gene editing therapies for human genetic diseases. **(A)** The yearly occurrences of top 10 keywords. **(B)** The cumulative occurrences of top 10 keywords over time. **(C)** Keyword co-occurrence network. **(D)** Top 25 keywords with the strongest citation bursts.

To elucidate the temporal evolution of research themes, Figures 7A, 7B illustrate the distribution of the top 10 keywords by occurrence frequency over time, where higher values denote greater frequency. The most prevalent keywords include “crispr/cas,” “gene editing,” “gene therapy,” “genome editing,” “sickle cell disease,” “zebrafish,” “induced pluripotent stem cells,” “disease models,” “cystic fibrosis,” and “base editing.” Figure 7C presents the keyword co-occurrence network, which delineates 11 distinct thematic clusters within the domain of gene editing therapies for human genetic diseases. Cluster 1 centers on stem cells in disease modeling and precision medicine, with high-frequency terms such as “induced pluripotent stem cells,” “disease models,” and “precision medicine.” Cluster 2 emphasizes artificial intelligence, ethics, and gene-editing governance, featuring “ethics,” “artificial intelligence,” and “machine learning.” Cluster 3 focuses on hemoglobinopathies and gene targeting, highlighted by “sickle cell disease,” “beta-thalassemia,” and “hematopoietic stem cells.” Cluster 4 addresses gene delivery, viral vectors, and clinical translation, with “gene editing,” “gene therapy,” and “viral vectors” as key nodes. Cluster 5 concentrates on induced pluripotent stem cells, cardiac and mitochondrial disorders, encompassing terms such as “induced pluripotent stem cells,” “genetics,” and “duchenne muscular dystrophy.” Cluster 6 explores AAV vectors, cystic fibrosis, and genomics, anchored by “cystic fibrosis,” “AAV vectors,” and “genomics.” Cluster 7 centers on next-generation editing tools: base and prime editing, with representative terms including “base editing,” “prime editing,” “talen,” and “zfn.” Cluster 8 focuses on CRISPR/Cas and DNA repair mechanisms, characterized by high-frequency terms such as “crispr/cas,”and “genome editing.” Cluster 9 addresses genetic regulation and long-read sequencing, featuring “gene regulation,” “familial hypercholesterolemia,” “ldlr,” and “long-read sequencing.” Cluster 10 concentrates on viral vectors for monogenic disorders, anchored by “lentiviral vector,” and“hemophilia b.” Cluster 11 serves as an overarching theme encompassing genetic disorders, capturing the broader conceptual framework that integrates the diverse therapeutic and mechanistic dimensions of gene editing applications.

Keyword burst analysis serves to identify sudden increases in the frequency of specific terms within defined time windows, thereby signaling emerging research frontiers. This approach effectively captures dynamic shifts in scientific focus within the field of gene editing for human genetic diseases. As illustrated in Figure 7D, the five keywords exhibiting the strongest burst strength are: “induced pluripotent stem cells” (strength = 6.81, 2016–2021), “non-homologous end” (strength = 6.55, 2008–2017), “cas9 system” (strength = 6.55, 2015–2018), “prime editing” (strength = 6.39, 2022–2023), and “base editing” (strength = 6.32, 2022–2023). Notably, keywords with bursts continuing through 2025 include “base editors” (strength = 4.47, 2020–2025) and “CFTR function” (strength = 4.03, 2022–2025).

## 4 Discussion

### 4.1 Main research directions and publication trends

Our bibliometric analysis of 1,571 publications from 2005 to 2025 underscores a paradigm shift in gene editing therapies for human genetic diseases, characterized by highly concentrated research activity and a clear translational trajectory. Hematologic disorders remain the vanguard of clinical application, with sickle cell disease and β-thalassemia emerging as the most extensively studied indications. This predominance is biologically grounded: hematopoietic stem cells (HSCs) are accessible for *ex vivo* manipulation, allowing for rigorous quality control and safety validation prior to reinfusion, thereby bypassing the complex barriers of *in vivo* delivery. In stark contrast, non-hematopoietic tissues face substantial technical barriers to delivery. These include immune clearance mechanisms that recognize and eliminate viral vectors and CRISPR components, as well as the necessity for tissue-specific promoters to achieve targeted expression without off-target effects. A deeper dive into these immunological and vector design challenges underscores the significant translational bottleneck beyond hematology. This advantage is evidenced by mature editing platforms and successful clinical translation, culminating in landmark regulatory approvals (e.g., Casgevy). While the field is rooted in genetics, molecular biology, and cell therapy—forming the mechanistic foundation for programmable nucleases—emerging engagement is expanding into *in vivo* delivery, precision editing tools, and non-hematopoietic disorders. However, these nascent areas remain limited in scale compared to blood-related research, highlighting a translational bottleneck rooted in tissue accessibility and delivery efficiency. Regarding publication dynamics, the field has followed a three-phase exponential growth trajectory: the era of engineered nucleases (2005–2012), the CRISPR revolution (2013–2018), and the precision translation era (2019–2025). A striking surge in output has occurred since 2019, with the past six years accounting for 77.3% of all publications, reflecting robust academic and industrial investment. Metrics including annual publication count, total citations, and usage confirm that the field has entered a period of accelerated expansion. Notably, a paradox exists between the high average citations of early foundational studies (2005–2012) and the lower average citations of recent publications (2023–2025). This discrepancy arises not from diminished scholarly value but from citation latency inherent to rapidly expanding fields: classic mechanistic works retain enduring influence through accumulative advantage, whereas newer clinical and applied studies have not yet accrued comparable impact. Furthermore, the dilution of average citation counts may also reflect the fragmentation of research into highly specialized sub-niches (e.g., specific delivery vectors or rare disease models), which naturally attract narrower audiences compared to foundational tool papers. This pattern is characteristic of a maturing therapeutic discipline transitioning from basic discovery to real-world application.

### 4.2 Major contributors and geopolitical landscape

Our analysis reveals a bipolar global structure in gene editing research, dominated quantitatively by the United States and China, which together contribute 47.2% of publications. However, this aggregate output masks profound qualitative divergences in their research ecosystems. The United States maintains hegemony in high-impact science, evidenced by its publications receiving 2.91-fold higher average citations than China's (54.5 vs. 18.7). We used citations / (2025–publication year + 1) as annual citations.Even after adjusting for citation age (average annual citations per paper), the U.S. still achieved a 2.2-fold higher impact compared to China (9.8 vs. 4.5 citations/year), though this gap may partly reflect differences in paper types and journal impact (see Limitations section). To account for temporal bias, we compared age-adjusted citation rates (average annual citations) rather than raw totals. However, field differences (methods vs. clinical papers) remain a limitation. This citation advantage may reflect a mature innovation paradigm potentially characterized by concentrated excellence. Hypothesized mechanisms include public-private synergy, though bibliometric data alone cannot establish causality (35). The distinct funding ecosystems shape this landscape: the U.S. system is underpinned by investigator-driven federal grants (e.g., NIH's 3.2 billion for genomics) and a robust venture capital ecosystem; China's output is driven by centralized, state-coordinated programs like the National Key R&D Program focused on strategic capacity building; while European nations benefit from coordinated, excellence-based funding mechanisms such as the European Research Council (ERC), fostering a quality-oriented research culture. These different funding models provide a deeper contextualization for the dominance and output profiles of specific institutions. Moreover, sustained federal investment, such as the NIH's $3.2 billion allocation for genomics in FY2023, enables high-risk, high-reward research (29). Crucially, the U.S. ecosystem benefits from a “flywheel effect,” where successful exits from biotech startups reinvest capital into early-stage innovation, sustaining the innovation cycle. In contrast, China demonstrates the most rapid expansion globally, with publication volume increasing 226-fold from 2012 to 2025, underpinned by massive institutional capacity building and centralized resource allocation (36). While China ranks second in publication count, the gap in average citation impact suggests a critical need to pivot from quantitative expansion to qualitative elevation, focusing on originality and clinical depth. Bridging this gap will require not only increased funding but also fostering a culture that rewards high-risk, original discovery over incremental follow-up studies. European nations, alongside Japan and South Korea, present a third model: moderate output volume but the highest citation efficiency (avg. 28.4), reflecting a sustained emphasis on mechanistic depth (37), robust ethical integration (38, 39), and perhaps more conservative, selectivity-driven research investment (40). Institutional analysis reveals strong concentration and a distinct Matthew effect, where a minority of elite institutions drive the majority of high-impact work. Harvard University, the University of California system, and Stanford University form the leading academic hub, with 12 of the top 15 most productive institutions based in the United States. Chinese institutions, led by the Chinese Academy of Sciences, demonstrate rapidly growing capacity within a centralized model. Nevertheless, most institutions remain in growth or exploratory phases with limited cross-border collaboration; networks remain predominantly national rather than globally integrated. This fragmentation poses a risk to global progress, as complex challenges like immune responses to editors require diverse, multinational clinical data. Future progress may depend on breaking these silos through international consortia similar to the Human Genome Project. The underlying reasons for this limited global integration, however, remain underexplored and are likely multifactorial, including funding disparities across nations, language barriers for non-Anglophone researchers, and geopolitical factors that shape scientific collaboration. Without incorporating such qualitative interpretation, the bibliometric finding remains superficial. Therefore, future mixed-methods studies are needed to systematically disentangle these drivers. Journal distribution reflects both dispersion and core concentration, with knowledge flows centering on molecular biology, genetics, and clinical medicine, anchoring the field's interdisciplinary identity.

### 4.3 Technological evolution and developmental history

The field's intellectual trajectory is demarcated by three sequential waves, each defined by a core technological paradigm. The foundational wave (2005–2012) of programmable nucleases (ZFNs, TALENs) established the concept but was limited by engineering complexity (2, 3, 41, 42). While ZFNs and TALENs have been largely superseded for mainstream applications, they still play specific and valuable roles in certain niche applications (e.g., targeting unique genomic loci in rare disease models), ensuring the model is accurate rather than oversimplified. Early studies focused on homologous recombination, double-strand break repair, and proof-of-concept gene correction in model systems. The conceptually delineated second wave (2013–2018) was triggered by CRISPR-Cas9 (34, 43–45), catalyzing exponential growth, a $6.8 billion venture capital influx (46), and the expansion of the Cas protein toolbox (47, 48). This breakthrough democratized gene editing, triggering early translational efforts toward *ex vivo* therapy. We are now firmly within the third wave (2019–2025), defined by a shift toward precision and clinical translation. This phase is dominated by base and prime editing technologies (10, 11, 32, 49, 50), signaling their transformative potential. Landmark clinical approvals for sickle cell disease and β-thalassemia validated the curative potential of *ex vivo* CRISPR therapy. Concurrently, *in vivo* editing emerged as a frontier, supported by advances in lipid nanoparticles (LNPs) and AAV vectors. A critical bottleneck and concurrent frontier within this wave is *in vivo* delivery (12, 51–53), representing a necessary convergence with materials science and nanotechnology to bridge the longstanding gap between molecular discovery and systemic therapeutic application (53, 54). Research priorities have shifted decisively from tool development to therapeutic safety, delivery efficiency, long-term efficacy, and real-world clinical implementation. Notably, the transition between phases is not merely chronological but represents a fundamental shift in risk profile: from proving feasibility to managing clinical safety and manufacturing scalability.

### 4.4 Current research hotspots, ethics, and future directions

Temporal analysis of keyword co-occurrence and citation bursts delineates distinct, time-dependent research hotspots. Early research trajectories focused primarily on homologous recombination and first-generation nucleases. The post-2013 era was characterized by the dominance of terms such as “CRISPR,” “Cas9,” and “stem cells,” reflecting widespread tool adoption. Since 2020, the most significant citation bursts have been associated with base editing and prime editing, marking a paradigm shift toward ultra-precise, double-strand break-free genome modification. To move beyond a qualitative mention, we conducted a comparative growth trajectory analysis: the average annual publication growth rate for base editing and prime editing studies was approximately 35% during 2020–2025, significantly outpacing the overall field's growth rate (27.7%) and confirming their status as accelerating research frontiers. While hematologic diseases remain persistent focal points, *in vivo* delivery systems have emerged as critical bottlenecks. From a translational perspective, the development pathway clearly mirrors technological maturity. *Ex vivo* editing strategies (8, 9, 55–57) have demonstrated established clinical viability and high translational value. Conversely, *in vivo* editing constitutes the next critical frontier. Although pioneering trials in liver and eye diseases prove feasibility (54), challenges such as pervasive immune responses (58), persistent off-target concerns (59), and limited tropism beyond hepatic tissues underscore that efficient, tissue-specific delivery remains the paramount obstacle to broadening therapeutic impact. Notably, our co-word analysis reveals a pronounced underrepresentation of ethical discourse within the mainstream scientific literature. The keyword “ethics” appears in only 1.8% of all papers, and its co-occurrence with “germline editing” is minimal (2.2%), far below expected levels for such a consequential area. This bibliometric disconnect is gravely underscored by real-world consequences, as seen in the 2018 He Jiankui incident (60). Furthermore, citation patterns reveal a disparity in utilization: ethics literature is cited more frequently in policy documents than in primary research, suggesting divergent perceptions of its utility between communities (25, 39). To bridge this gap, we propose systemic interventions: mandating ethical consideration sections in high-impact journals, integrating bioethicists as co-investigators on major grants, developing “ethics impact” metrics, and strengthening enforceable international governance frameworks (61, 62). As the technology approaches widespread clinical deployment, bridging this “ethics gap” is imperative to prevent public backlash and ensure equitable access. Integrating these insights with burst analysis, funding trends, and trial data, we forecast five strategic priorities for future development (29): (1) **Advanced Precision Editing**, involving the continued optimization of base editors, prime editors, and epigenome modifiers to expand targetable mutations without inducing double-strand breaks; (2) *In Vivo* **Delivery Breakthroughs**, including the development of compact nucleases (e.g., Cas12f) to overcome delivery vehicle constraints(63–65), alongside tissue-selective vectors and non-viral systems; (3) **Expanded Disease Indications**, signifying a significant expansion beyond hematology to oncology, monogenic metabolic disorders, neurodegenerative, cardiovascular, and regenerative applications(8, 9, 52, 53, 66–70); (4) **AI-Assisted Design**, integrating machine learning for guide RNA prediction, off-target assessment, and personalized therapeutic design; and (5) **Ethical and Equitable Translation**, strengthening governance and global collaboration to address disparities and ensure gene editing benefits all of humanity.

## 5 Strengths and limitations

While this study maps the evolutionary trajectory of gene editing therapies for human genetic diseases through bibliometric methods, several limitations should be kept in mind. First, bibliometrics captures macro-trends in scientific output but cannot substitute for the rigorous evidence appraisal of systematic reviews or meta-analyses; it traces publication surges without addressing the methodological quality or clinical efficacy of individual studies. Second, our exclusive reliance on Web of Science Core Collection and Scopus, together with an English-only filter, may introduce selection and linguistic biases, potentially overlooking relevant research published in regional or non-indexed venues. A further caveat concerns the search strategy itself. By centring on “gene editing” in explicitly therapeutic contexts, our query may have selectively missed foundational studies on delivery vehicles, off-target detection platforms, and long-term safety profiling whose primary keywords do not routinely include “therapy” or “genetic disease”. We acknowledge that quantifying citation half-life or thematic specialization would provide a stronger argument for journal centrality and impact. Therefore, we explicitly clarify that our interpretation of low link strength as a peripheral position is provisional and based on structural network metrics rather than a temporal impact dynamic like citation half-life. As a result, the contribution of these enabling technologies to clinical translation is likely underrepresented in our co-citation and keyword maps, and the bibliometric landscape depicted here should be regarded as the therapeutic translation front as seen from the vantage point of editing-focused literature rather than a complete inventory of every translational building block. Furthermore, we explicitly acknowledge that our study does not substantiate the claim regarding ‘equitable access' with direct bibliometric evidence, such as geographic distribution of clinical trials or patent ownership. This constitutes a key limitation, as our data does not allow for a systematic analysis of equity dimensions. Additionally, we explicitly acknowledge a citation window bias as a key limitation: the observed declining average citation counts for recent publications are largely an artifact of their limited citation windows rather than a reflection of reduced scholarly impact. We explicitly acknowledge this limitation to avoid inadvertently implying a decline in the value of more recent works. We did not perform full field-normalized citation normalization due to data constraints, which may affect cross-disciplinary comparisons. Notwithstanding these constraints, the study offers distinct strengths. By leveraging visual clustering algorithms, we transform complex bibliographic data into intuitive maps of research hotspots and paradigm shifts, furnishing a macroscopic perspective that can guide future innovation in CRISPR-based therapies, off-target mitigation, and delivery systems.

## 6 Conclusions

This comprehensive bibliometric analysis, spanning 1,571 publications from 2005 to 2025, delineates the trajectory of gene editing from a foundational molecular tool to a clinical therapeutic paradigm. The field exhibits a bipolar global structure dominated by the United States and China, yet is defined by three distinct technological waves culminating in the current era of precision editing and *in vivo* delivery. While translational breakthroughs have been achieved, a critical bottleneck in delivery technology and a profound ethics-research integration gap emerge as the principal constraints on the field's potential. To navigate this inflection point, future progress necessitates a concerted focus on overcoming delivery limitations, expanding therapeutic indications through prime and epigenetic editing, and implementing systemic measures—from mandated ethical frameworks to harmonized global governance—to ensure the responsible realization of gene editing's transformative promise.

## Data Availability

The original contributions presented in the study are included in the article/supplementary material, further inquiries can be directed to the corresponding authors.
