# Supplementary material for: The evolving landscape of gene editing therapies for human genetic diseases: a twenty-year bibliometric analysis
Source: Front Med (Lausanne). 2026 Jun 3;13:1872028. doi: 10.3389/fmed.2026.1872028 (PMC13272081; doi:10.3389/fmed.2026.1872028)

### **A1. Deduplication Protocol**

To ensure data integrity, a systematic deduplication process was applied to the merged dataset from Web of Science Core Collection (WoSCC) and Scopus.

**1. Raw Data Retrieval:**

WoSCC: 1133 records

Scopus: 614 records

**Total raw records before merging**:1747 records

**2. Merging and Initial Deduplication:**
Records from both databases were imported into Python (version 3.9) and merged based on their unique identifiers (DOI, PubMed ID, or similar). The initial deduplication step used exact matching on the following fields:

**DOI** (preferred)

**Title** (case-insensitive exact match)

**First Author + Publication Year** (combined)

**3. Fuzzy Matching (Secondary Deduplication):**
After exact matching, a fuzzy matching step was applied to identify highly similar records where metadata variations may have prevented exact matching (e.g., minor title differences, trailing spaces). The following parameters were used:

**Software**: fuzzywuzzy (Python package)

**Fields compared:** Title (pre-processed by removing punctuation and converting to lowercase)

**Similarity threshold:** 85 (using Levenshtein distance ratio)

**Action:** Records with similarity ≥ threshold were manually reviewed and consolidated.

**4. Screening Process:**

**Records after deduplication:** 1649

**Records excluded during title/abstract screening:** 63 (Reasons: non-therapeutic context, purely computational studies, non-primary document types)

**Records excluded during full-text screening:** 15 (Reasons: incomplete metadata, retracted publications)

**Final included records for analysis:** 1,571

**5. Exclusion Reasons Summary:**non-therapeutic context, purely computational studies, non-primary document types, incomplete metadata, retracted publications

### **A2. Software Parameter Justification**

**1. CiteSpace (version 6.4.R1)**

**Time slicing:** 1 year per slice (2005–2025). This interval was chosen to balance temporal resolution with network stability.

**Top N per slice:** 50 (Top N most cited or co-occurring items per year). This value was selected to ensure sufficient node inclusion while avoiding overly sparse networks.

**Pruning algorithm:** Pathfinder scaling + Pruning Sliced Networks. Pathfinder scaling was selected over Minimum Spanning Tree (MST) or Clustering (MCL) because it removes more non-essential links while preserving the structural integrity of the network, reducing noise without over-simplifying. Pruning Sliced Networks further refines the network by removing weak links within each time slice.

**Node selection:** Keywords, Cited References, Authors (dependent on analysis).

**2. VOSviewer (version 1.6.20)**

**Normalization method:** Association strength (LinLog/modularity). This method was chosen because it is appropriate for weighted networks and is less affected by skewed degree distributions compared to standard cosine or correlation measures.

**Clustering resolution:**  (default).

**Minimum cluster size:** 5.

**Minimum document thresholds (co-authorship analysis):**

Authors: 5

Institutions: 20

Countries: 50

**Layout:** Attraction:2 , Repulsion: 1.

### **A3. Summary Table of Analytical Parameters**

| Analysis | Software | Key Parameter | Value |
| --- | --- | --- | --- |
| Co-authorship network | VOSviewer | Minimum documents (Authors) | 5 |
| Institutional network | Scimago Graphica | Minimum documents (Institutions) | 20 |
| Country collaboration | R studio | Minimum documents (Countries) | 50 |
| Keyword co-occurrence | VOSviewer | Normalization | Association strength |
| Burst detection | CiteSpace | Time slice | 1 year |
| Network pruning | CiteSpace | Algorithm | Pathfinder scaling |
| Clustering | VOSviewer | Resolution | default |


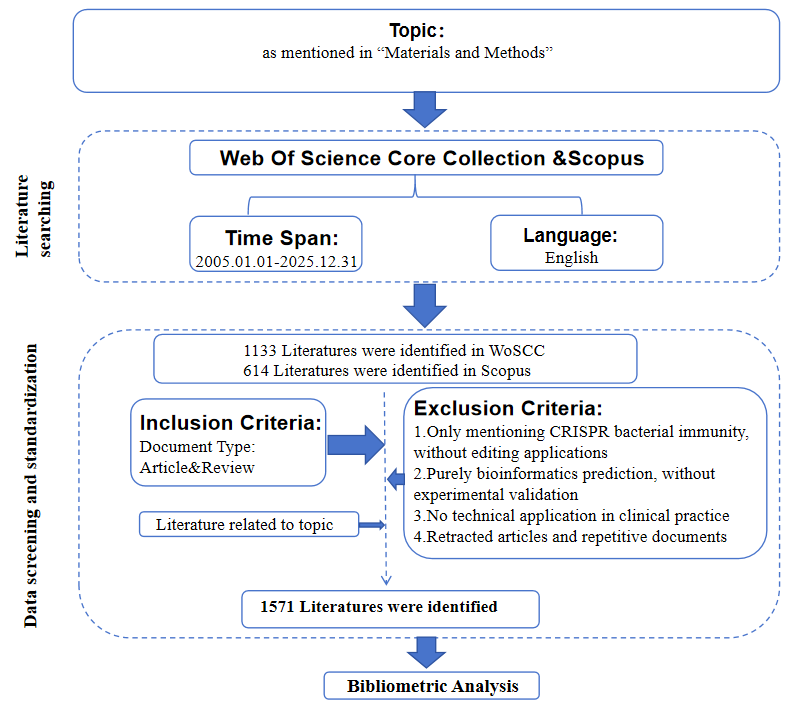

Supplement: Supplementary file 1 [file Supplementary_file_1.docx]
