# Supplementary material for: The evolving landscape of gene editing therapies for human genetic diseases: a twenty-year bibliometric analysis
Source: Front Med (Lausanne). 2026 Jun 3;13:1872028. doi: 10.3389/fmed.2026.1872028 (PMC13272081; doi:10.3389/fmed.2026.1872028)
Supplement: Supplementary file 2 [file Table_1.docx]

**Supplementary Table 1.** Strategies in the Web of Science Core Collection

| **Step** | **Formular** |
| --- | --- |
| #1 | TS = ("gene editing" OR "genome editing" OR "CRISPR" OR "CRISPR-Cas9" OR "CRISPR/Cas9" OR "Cas9" OR "Cas12" OR "Cas13" OR "ZFN" OR "zinc finger nuclease" OR "TALEN" OR "transcription activator-like effector nuclease" OR "base editing" OR "base editor" OR "prime editing" OR "prime editor" OR "homology-directed repair" OR "HDR" OR "non-homologous end joining" OR "NHEJ" OR "programmable nuclease") |
| #2 | TS=("genetic disease*" OR "genetic disorder*" OR "inherited disease*" OR "hereditary disease*" OR "monogenic disease*" OR "autosomal dominant" OR "autosomal recessive" OR "X-linked" OR "sickle cell disease" OR "beta-thalassemia" OR "cystic fibrosis" OR "Duchenne muscular dystrophy" OR "Huntington's disease" OR "Familial hypercholesterolemia" OR "Spinal muscular atrophy" OR "Tay-Sachs disease" OR "Phenylketonuria" OR "Li-Fraumeni syndrome" OR " Y-linked") |
| #3 | TS=("human*" NOT "animal*" NOT "mouse" NOT "mice" NOT "rat" NOT "rat model") |
| #4 | #1 AND # 2 AND #3 |

**Supplementary Table 2 .** Search Strategies in the Scopus

| **Formular** |
| --- |
| TITLE-ABS-KEY ( "gene editing" OR "genome editing" OR "CRISPR" OR "CRISPR-Cas9" OR "CRISPR/Cas9" OR "Cas9" OR "Cas12" OR "Cas13" OR "ZFN" OR "zinc finger nuclease" OR "TALEN" OR "transcription activator-like effector nuclease" OR "base editing" OR "base editor" OR "prime editing" OR "prime editor" OR "homology-directed repair" OR "HDR" OR "non-homologous end joining" OR "NHEJ" OR "programmable nuclease" ) AND ( "genetic disease*" OR "genetic disorder*" OR "inherited disease*" OR "hereditary disease*" OR "monogenic disease*" OR "autosomal dominant" OR "autosomal recessive" OR "X-linked" OR "sickle cell disease" OR "beta-thalassemia" OR "cystic fibrosis" OR "Duchenne muscular dystrophy" OR "Huntington's disease" OR "Familial hypercholesterolemia" OR "Spinal muscular atrophy" OR "Tay-Sachs disease" OR "Phenylketonuria" OR "Li-Fraumeni syndrome" OR " Y-linked" ) AND ( "human*" NOT "animal*" NOT "mouse" NOT "mice" NOT "rat" NOT "rat model" ) |
